# Novel Bio-Functional *Aloe vera* Beverages Fermented by Probiotic *Enterococcus faecium* and *Lactobacillus lactis*

**DOI:** 10.3390/molecules27082473

**Published:** 2022-04-12

**Authors:** Ruth B. Cuvas-Limón, Pedro Ferreira-Santos, Mario Cruz, José António Teixeira, Ruth Belmares, Clarisse Nobre

**Affiliations:** 1 Food Research Department, School of Chemical Sciences, Autonomous University of Coahuila, Saltillo Coahuila, Boulevard Venustiano Carranza e Ing. José Cárdenas s/n Col. República C.P., Saltillo 25280, Mexico; rcuvas@uadec.edu.mx; 2 CEB—Centre of Biological Engineering, University of Minho, Campus de Gualtar, 4710-057 Braga, Portugal; pedrosantos@ceb.uminho.pt (P.F.-S.); jateixeira@deb.uminho.pt (J.A.T.); 3 LABBELS—Associate Laboratory, 4710-057 Braga, Portugal; 4 Department of Food Science and Technology, Antonio Narro Autonomous Agricultural University, Calzada Antonio Narro, No. 1923 Col. Buena Vista C.P., Saltillo 25315, Mexico; mike80@hotmail.com

**Keywords:** *Aloe vera*, lactic acid bacteria, phenolic compounds, antioxidant activity, organic acids, functional food

## Abstract

*Aloe vera* has been medicinally used for centuries. Its bioactive compounds have been shown to be very effective in the treatment of numerous diseases. In this work, a novel functional beverage was developed and characterized to combine the health benefits of probiotic bacteria with the *Aloe vera* plant itself. Two *Aloe vera* juices were obtained by fermentation either by a novel isolated *Enterococcus faecium* or a commercial *Lactococcus lactis*. The extraction of *Aloe vera* biocompounds for further fermentation was optimized. Extraction with water plus cellulase enhanced the carbohydrates and phenolic compounds in the obtained extracts. The biotransformation of the bioactive compounds from the extracts during fermentation was assessed. Both probiotic bacteria were able to grow on the *Aloe vera* extract. Lactic acid and short-chain fatty acids (SCFA) together with fourteen individual phenolic compounds were quantified in the produced *Aloe vera* juice, mainly epicatechin, aloin, ellagic acid, and hesperidin. The amount of total phenolic compounds was maintained through fermentation. The antioxidant activity was significantly increased in the produced juice by the ABTS method. The novel produced *Aloe vera* juice showed great potential as a functional beverage containing probiotics, prebiotics, SCFA, and phenolic compounds in its final composition.

## 1. Introduction

The food industry has been focused on the development of functional food products, i.e., food that can beneficially affect specific functions of the human body beyond adequate nutritional effects, leading to improved health and well-being and/or reduced risk of disease [1]. Functional food products are manufactured through the addition of exogenous natural compounds, probiotics, or other microorganisms able to produce biogenic compounds [2]. Regarding functional foods that include probiotic microorganisms, non-dairy fermented drinks appear as beverages of great interest, as they represent an alternative for specific consumers, such as lactose intolerants and vegetarians, among others [3].

Many functional foods produced on a large scale have probiotic microorganisms incorporated due to their numerous health benefits, including antimicrobial, antimutagenic, anticarcinogenic, and antihypertensive effects, as well as reduction of serum cholesterol, allergic symptoms and diarrhea, lactose intolerance alleviation, and stimulation of the immune system [2]. The majority of the probiotic microorganisms belong to lactic acid bacteria (LAB), including *Lactobacillus*, *Lactococcus*, *Enterococcus* and *Bifidobacteria* [3,4]. These microorganisms are resistant to low pH and easily adapt to different substrate sources [5].

Phenolic compounds are widely present in fruits and plants as well as their by-products. Thus, they are the most important group of natural antioxidants in the diet. These bioactive molecules act as reducing agents and can improve human health by preventing or treating common diseases and conditions, such as obesity, type 2 diabetes, hypertension, and metabolic syndrome [6]. However, the present understanding of its biological effects is yet to be unveiled, such as anti-inflammatory activity, stimulation of microbial growth, and their impact on the production of beneficial metabolites by different microorganisms, especially probiotics [7].

The interaction between probiotics and phenolic compounds may increase the antioxidant activity of the final products. During the probiotic fermentation process, the division/dissociation of the bonds between phenolic compounds and other constituents is facilitated. This leads to the release of simple phenolic compounds, which increases antioxidant capacity [8,9]. Thus, the combination of LAB with natural antioxidants can open new biotechnological opportunities for the expansion of the current functional beverages market.

Since plants are an important source of bioactive molecules, the incorporation of probiotics in plant-based beverages may increase their nutritional and functional value. One plant that has gained great popularity due to its medicinal characteristics is *Aloe vera*. *Aloe vera* is a plant belonging to the *Asphodelaceae* group. Many biologically active compounds have been identified in the plant. Thus, *Aloe vera* has been applied to wounds and inflammations and be used in the treatment and prevention of several diseases, namely cancer, diabetes, ulcer, microbial and skin diseases, and acquired immune system deficiency syndrome [10].

Currently, *Aloe vera* has been used in the development of differentiated products with functional potential, such as beverages. *Aloe vera* contains in its composition polysaccharides, proteins, minerals, phenolics, anthraquinones, enzymes, and amino acids [11]. Its chemical composition makes it an excellent substrate for microorganisms’ growth, such as probiotics.

The present work aims at producing an innovative functional beverage with antioxidant potential and high-value bioactive compounds based on *Aloe vera* fermentation by a novel isolated probiotic LAB.

## 2. Results and Discussion

*Aloe vera* has been used for centuries due to its therapeutic benefits, in addition to being used in the food industry for the development of new functional products. Fillet samples of *Aloe vera* cultivated in the same geographical conditions as the one used in this study (Ibiza, Spain) were characterized by other authors with the following composition: water 98 g moisture/100 g in the gel of *Aloe vera* (the major component), protein 0.044 g/100 g, ash 0.450 g/100 g, fat 0.01 g/100 g and carbohydrates 0.630 g/100 g [12]. Similar results were reported by other authors [13,14]. Herein, an *Aloe vera* extract was used to produce a symbiotic juice obtained by fermentation with probiotic bacteria. The *Aloe vera* extraction process was firstly optimized. Before and after fermentation, *Aloe vera* extract and the produced juice were characterized in their content of carbohydrates, organic acids, phenolic compounds, aloin, and antioxidant activity. Results are further shown and discussed.

### 2.1. Aloe vera Extracts Preparation and Characterization

The parenchyma cells contain a transparent mucilaginous jelly which is referred to as *Aloe vera* gel. The *Aloe vera* gel was homogenized and further extracted from the plants until a more liquid but viscous consistency was obtained.

Subsequently, the *Aloe vera* lyophilized gel was submitted to four different solid–liquid extractions to recover its bioactive compounds, namely, water, water plus cellulase, 80% ethanol:water, and 80% ethanol:water plus cellulase. The extraction yield is clearly influenced by the type of solvent used in the extraction process. The results obtained showed that the water plus cellulase was the best solvent for recovering the biocompounds from *Aloe vera* gel (96 ± 3%) followed by water (92 ± 3%). Ethanol showed lower extraction efficiency with yields of around 52% regardless of the combined use with cellulase.

**Figure 1:**
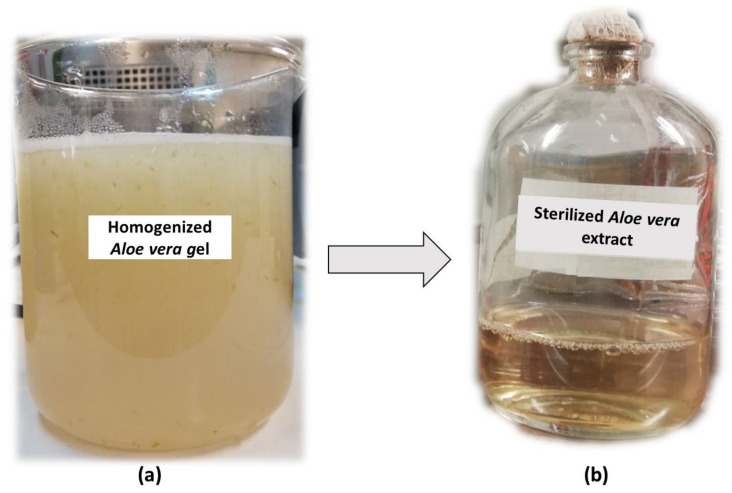
*Aloe vera* used in this work: (**a**) homogenized *Aloe vera* gel, (**b**) filtered and sterilized *Aloe vera* extract.

The liquid obtained after the extraction, regardless of the solvent used, was more transparent and less viscous than the original gel, as shown in Figure 1.

Cellulase was herein applied to break down the cellulose and the small amounts of hemicellulose and lignin (insoluble fiber) of the *Aloe vera*, reducing the viscosity of the extract and facilitating the recovery of bio-functional compounds with industrial interest. The insoluble fiber fraction may correspond to the cell walls of the parenchyma cells contained in the gel [12]. Using water extraction, the polymers that are not cross-linked in the cell wall network are solubilized, extracting mannose-rich polysaccharides [14]. The filtration step applied allowed removing the gel fibrous material and simultaneously sterilizing the *Aloe vera* extract before the fermentation step, which is critical to maintaining the quality of the resulting fermented products.

#### 2.1.1. Carbohydrates in the Extracts

As previously mentioned, *Aloe vera* extracts were obtained by the application of different solvents. Analyzing the profile of the individual monosaccharides obtained in each extract, it is observed that a higher amount of glucose is extracted when cellulase is applied, i.e., water plus cellulase (4058.12 ± 15.10 mg/L) and ethanol plus cellulase (4379.96 ± 14.88 mg/L). Regarding mannose and galactose, a much higher concentration (approximately 50%) was determined in the aqueous extractions, with water (7353 ± 1 mg/L) and water plus cellulase (7312 ± 6 mg/L) rather than in the ethanol extractions. Mannose and galactose co-eluted; therefore, it was not possible to obtain the individual concentration of these sugars. Arabinose was not detected in the extraction carried out with water without cellulase. However, the highest concentration of arabinose was obtained when adding cellulase to the water (4009.19 ± 1.47 mg/L), even when compared with the extraction carried out with ethanol. Fructose was detected in all extracts but at a trace amount.

The polysaccharides content identified in the *Aloe vera* includes mainly acemannan (50%), cellulose (25%), pectic polysaccharides (20%), and xylose-containing polysaccharides (5%) [15]. *Aloe vera* mannan is acetylated at the C-2 and C-3 positions and contains some side chains, mainly of galactose attached to C-6. The main monosaccharides identified in *Aloe vera* were glucose and mannose representing between 55 and 75% of the total monosaccharides determined [14,15,16]. Other sugars that have been reported in *Aloe vera* are rhamnose, fucose, arabinose, xylose, galactose, and uronic acids [14]. Many biological activities have been associated with acemannan, such as immunoregulation, anti-cancer, anti-oxidation, wound healing and bone proliferation promotion, neuroprotection, antiviral activity, immunomodulation, reduction of opportunistic infections, and intestinal health promotion, among others [13,17]. Ramified fructans have also been extracted from *Aloe vera* [17]. Both fructans and acemannan from *Aloe vera* have been identified as very promising prebiotics since they stimulated in vitro the growth of probiotic strains during fermentation with human stool samples [17].

Enzymatic methods have been usually applied for sugar extraction. In addition, treatment with enzymes reduces the viscosity of the solutions obtained from the *Aloe gel*, which facilitates its processing. Concomitantly, enzymes such as cellulase hydrolyze the *Aloe vera* polysaccharides, reducing their molecular size [18]. Cellulase hydrolyzes the polymer bonds, releasing its monomers, such as glucose, mannose, arabinose, xylose, fructose and fucose, which justifies the results herein obtained [14,19]. By applying cellulase to the *Aloe vera*, an increase in the concentration of all monosaccharides in the extracts was obtained. As monosaccharides are the carbon source used by probiotic bacteria for growth during fermentation, their application during the *Aloe vera* extraction seems to be of great importance.

#### 2.1.2. Phenolic Compounds in the Extract

Results from the TPC obtained by the method of Folin–Ciocalteu showed that the highest amount of phenolics was observed in the extracts where water plus cellulase was used (479 ± 12 mg GAE/L), which was followed by ethanol plus cellulase (469 ± 4 mg GAE/L). There were no significant differences (*p* > 0.05) between the amount of TPC obtained by extraction with only water or ethanol (442 ± 3 and 442 ± 1 mg GAE/L, respectively).

The enzymatic-assisted extraction with cellulase seems to influence (positively) the phytochemical profile of *Aloe vera* extracts, both in terms of phenolic compounds as well as carbohydrates. This may be explained by the increasing amount of sugars released when the cellulase is applied. The *Aloe vera* gel contains phenolic compounds that can be soluble free or conjugated soluble and insoluble. Conjugated soluble phenolic compounds bind soluble molecules such as carbohydrates, proteins, and lipids by esterification in the carboxylic moiety or etherification in the hydroxyl group. Insoluble phenolic compounds are generally covalently bound to polymers such as polysaccharides and lignins through an ester bond, and they are only released from the matrix through acidic, alkaline, or enzymatic hydrolysis [20].

Polyphenols are secondary plant metabolites whose structure includes one or more aromatic rings substituted by one or more hydroxyl groups and are strong antioxidants. Therefore, phenolics are combined with sugars, such as glucose, galactose, arabinose, ramose, and xylose [21]. Phenolic compounds are multifunctional and can act as reducing agents, hydrogen-donating antioxidants, and singlet oxygen quenchers [22]. Their potential as antioxidant compounds encourages their use in the prevention and treatment of various diseases associated with oxidative stress, such as cancer, cardiovascular diseases, inflammation, and others. However, the chemical structure of polyphenols can affect their bioavailability [23,24].

**Figure 2:**
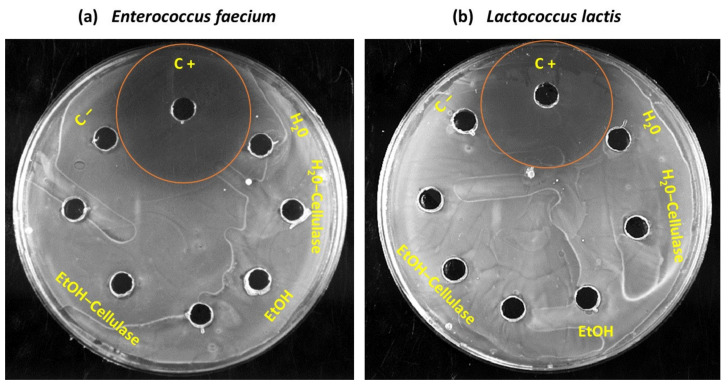
Evaluation of growth inhibition factors of (**a**) *Enterococcus faecium* and (**b**) *Lactococcus lactis* in *Aloe vera* extracts. C+ = Positive control; C− = Negative control; EtOH = Ethanol.

Furthermore, tests of inhibitory activity of the *Aloe vera* extracts in *E. faecium* and *L. lactis* showed that the extracts obtained by the different methods (after evaporation of ethanol) did not inhibit or modify the growth of both bacteria (Figure 2). Microorganisms inhibition was only observed in the control assay with ampicillin (C+). *E. faecium* and *L. lactis* showed resistance to ampicillin (antibiotic).

Since the extraction with water plus cellulase has been shown to enhance the amount of sugars and phenolic compounds in the obtained extracts, this extract was selected for further production of the functional *Aloe vera* juice by fermentation with *E. faecium* and *L. lactis*.

### 2.2. Production of the Aloe vera Juice

*E. faecium* has had a breakthrough in the food industry. Enterococci are important for fermentation and contribute to the ripening and aroma development of certain fermented cheeses and sausages. They are also used as probiotics to improve human or animal health.

Enterococci are Gram-positive bacteria, catalase-negative, cocci, facultative anaerobes and can grow at temperatures between 10 and 45 °C and pH 4 and 9.6 [25,26,27]. Enterococci have been applied to food to develop the organoleptic properties of fermented foods [28]. They have been used as starter cultures, improving biochemical properties, contributing to carbohydrate metabolism, and promoting the production of volatile compounds. They also enhance the ability to produce bioactive peptides and function as effective probiotics [27]. For this reason, *E. faecium* isolated from human breast milk was selected as the probiotic microorganism to ferment the *Aloe vera* extract obtained with water plus cellulase.

On the other hand, *L. lactis* has been used for centuries as a starter culture of fermented products, especially dairy products. *L. lactis* is therefore generally recognized as safe (GRAS) by the Food and Drug Administration (FDA) [29]. *Lactococcus* is classified as a Gram-positive, spherical, homolactate, non-sporulating, and facultative anaerobic gut bacteria. *L. lactis* belongs to the LAB group and therefore produces acid that promotes food preservation. It also produces bacteriocins, which improve food preservation, reinforcing its role in the food industry [30]. Moreover, *L. lactis* improves the flavor of fermented foods. For these reasons, a commercialized *L. lactis* was herein used to ferment the *Aloe vera* extract obtained with water plus cellulase. Results were further compared with fermentation run with the new isolated *E. faecium*, as shown below.

**Figure 3:**
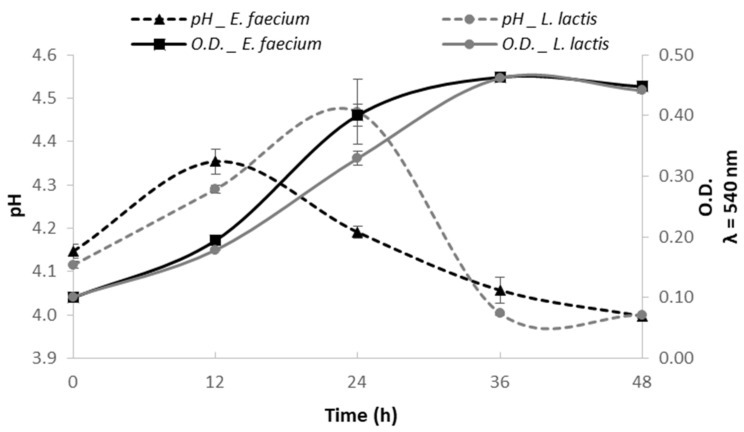
*Enterococcus faecium* (black) and *Lactococcus lactis* (gray) growth kinetics (full line) and pH profile (dashed line) in the *Aloe vera* extract.

Figure 3 shows the *Aloe vera* fermentation profile obtained with *E. faecium* and *L. lactis*.

Although the pH of the *Aloe vera* gel has been reported to be around 6.0 [31], the initial pH determined in the extracted juice from the gel using water plus cellulase showed to be around 4.1, meaning that the extraction process influenced the pH of the obtained juice. The pH of *E. faecium* initiated at 4.15 ± 0.02 and slightly increased up to 4.35 ± 0.03 at 12 h fermentation (*p* < 0.05). However, after 24 h, the pH started decreasing, reaching 4.06 ± 0.03 at the end of fermentation (48 h) (*p* < 0.05). The slight increase in the pH during the first 12 h may be related to the adaptability of *E. faecium* to the medium (*Aloe vera*). It has been reported that *E. faecium* grows adequately in a pH range of 4 to 9.6. However, *E. faecium* has shown to have the ability to create an acidic media, hence its probiotic potential [28].

Regarding the *Aloe vera* fermentation with *L. lactis*, the pH started at 4.12 ± 0.01, increased significantly up to 4.47 ± 0.01 at 24 h (*p* < 0.05), and then started decreasing to 4.003 ± 0.003, at 36 h fermentation. Between 36 and 48 h of fermentation, there were no significate changes in the pH (*p* > 0.05).

It has been reported that LAB are capable of lowering the pH of the medium in fermentation processes since they produce lactic acid in a reaction catalyzed by a lactic hydrogenase [32]. For both bacteria, the lowest pH obtained was 4.0, which characterized the final *Aloe vera* produced juice. The changes in pH are often used as indicators of fermentability and are related to the production of organic acids.

**Figure 4:**
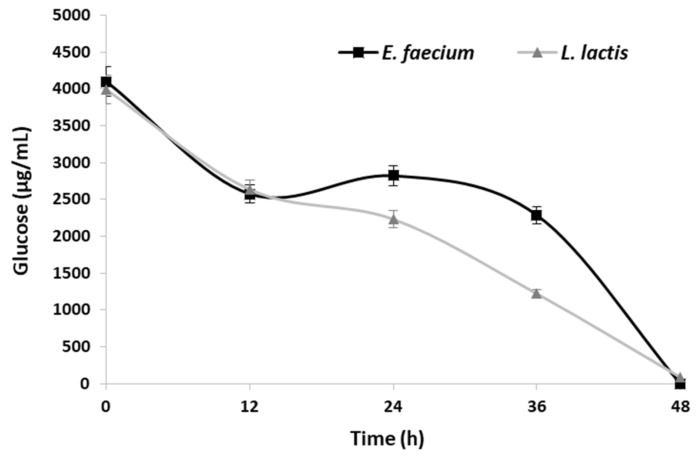
Profile of glucose during fermentation of the *Aloe vera* juice by *Enterococcus faecium* (black) and *Lactococcus lactis* (gray).

Results obtained showed that *Aloe vera* juice can be used as a propagation medium for both *E. faecium* and *L. lactis* due to its chemical composition. Both LAB grew on the *Aloe vera* extract obtained with water plus cellulase. A similar growth profile was achieved for both LAB, reaching its maximal growth at 36 h. The optical density of the fermented *Aloe vera* was kept constant between 36 and 48 h when the pH achieved values around 4.0. The low pH values may have contributed to the end of fermentation together with the depletion of glucose in the medium (Figure 4).

*Aloe vera* juice has also proven to be a good substrate for the growth of other probiotics. Although, to the best of our knowledge, the fermentation of *Aloe vera* with *E. faecium* or *L. lactis* has never been attempted. Other probiotic strains have been applied to *Aloe vera* juices from different varieties and cultivated under different agro-climatic/phyto-geographical regions. González et al. [31] evaluated the growth of *Lactobacillus plantarum* and *Lactobacillus casei* on an *Aloe vera* juice from the *barbadensis* variety, gown in Yucatán, México. Yagi et al. [33] demonstrated that *Aloe vera* juice certificated by International Aloe Science Council (IASC) could promote the growth of probiotic *Lactobacillus fermentum*. Nagpal et al. [34] studied the effect of *Aloe vera* juice on the growth and activity of probiotic *Lactobacilli*. After 48 h fermentation of a 100% *Aloe vera* juice with a *L. plantarum*, the pH decreased from 6.7 to 5.5, and for *Lactobacillus acidophilus*, it decreased from 6.2 to 5.8. In the same work, fermentations run in 100% MRS broth, using the same bacteria, showed a much higher decrease in the pH, reaching values around 4.6. The lower decrease in the pH values in *Aloe vera* indicates a slower growth of the bacteria in the plant. In this study, the initial pH was much lower (4.2), and therefore, the final pH was similar to the initial one (4.0), indicating also a slow growth of both *E. faecium* and *L. lactis* in the *Aloe vera* variety tested herein.

*Aloe vera* glucose was consumed during fermentation with *E. faecium* and *L. lactis*, as shown in Figure 4. At 48 h fermentation, *E. faecium* consumed the glucose in its totality, and only a trace amount of glucose was detected for the *L. lactis* fermentation (87 mg/L). Therefore, both bacteria were able to use *Aloe vera’s* carbohydrates as a substrate and source of carbon.

*E. faecium* are chemo-organotrophic bacteria that ferment sugars to produce mainly lactic acid. *Aloe vera* is a matrix mainly composed of non-digestible oligosaccharides, polysaccharides, and saccharides such as cellulose, glucose, mannose, L-rhamnose, and aldopentose [35]. In its nutritional composition, *Aloe vera* has primary carbohydrates, minerals, amino acids, and vitamins, which have been reported to successfully promote the growth of probiotic microorganisms [31].

### 2.3. Organic Acids in the Fermented Juice

**Figure 5:**
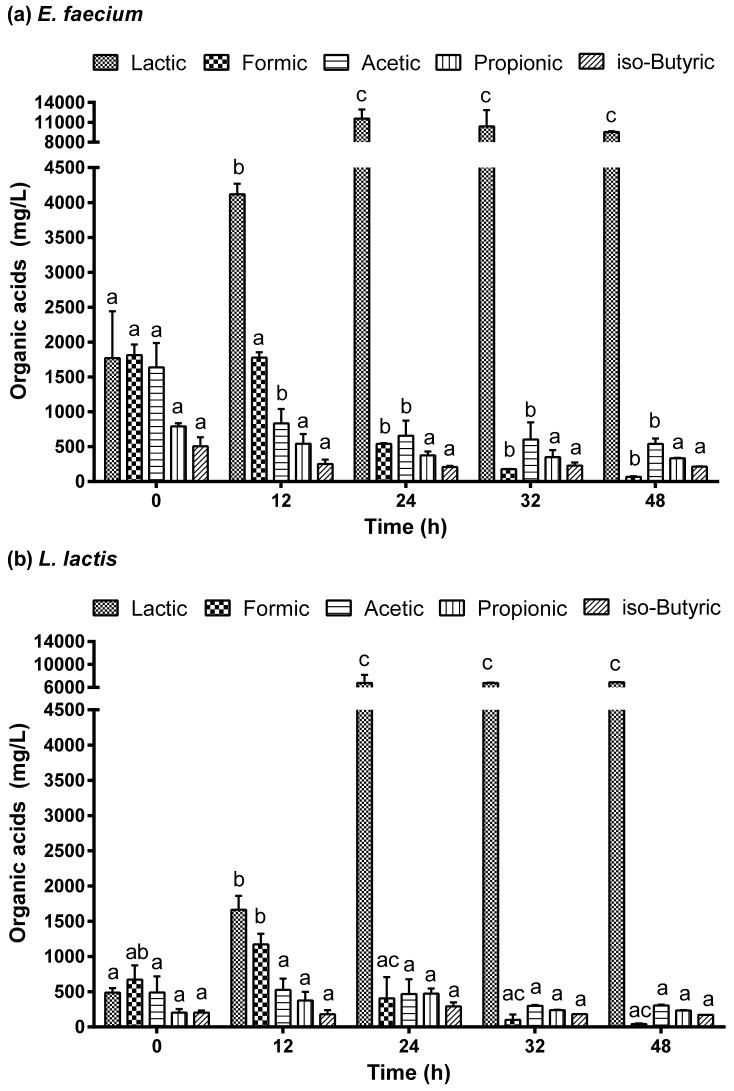
Organic acids profile during fermentation of *Aloe vera* extract with *Enterococcus faecium* (**a**) and *Lactococcus lactis* (**b**). Different letters a–c show significant differences (*p* < 0.05) between different fermentation times for the same organic acid.

The organic acids identified and quantified in the *Aloe vera* extract (0 h, fermentation) and along the fermentation with *E. faecium* and *L. lactis* are shown in Figure 5. A total of five organic acids were detected. Among the organic acids, lactic acid and several short-chain fatty acids (SCFA) were identified: namely, formic, acetic, propionic and iso-butyric acid. Formic, acetic and lactic acid were at the highest concentration, while propionic and iso-butyric acids were present in a lower amount. The profile of organic acids that characterized the initial *Aloe vera* extract used to produce the probiotic juice was: 752 ± 78 formic acid, 646 ± 4 acetic acid, 535 ± 48 lactic acid, 289 ± 83 propionic acid and 220 ± 14 mg/L of iso-butyric acid.

Some of these organic acids have been identified by other authors in *Aloe vera* from different sources [36,37]. Zhang et al. [37] identified eight organic acids in samples from fresh *Aloe vera* leaves from China and Mexico and in commercialized *Aloe vera* powder: namely, lactic, acetic, oxalic, L-malic, isocitric, isocitric acid lactone, citric and fumaric acid. The amount of each acid varied significantly according to the origin of the *Aloe vera* sample, the part of the plant used (e.g., whole fresh leaf, rind, gel), its preparation stage and the methods applied (e.g., freeze drying, spray drying, ethanol precipitation). Bozzi et al. [36] detected organic acids, such as lactic, acetic, formic, malic, citric, fumaric and succinic, in different commercialized samples of *Aloe vera* gel. Nejatzadeh-Barandozi [22] identified a number of organic acids in gel extracts from *Aloe vera* leaves harvested in September from farms in Iran, such as lactic, malic, glycolic, furoic, succinic, 2-methylsuccinic, tartaric, isonicotinic and 2-hydroxybutyric. The organic acids composition of *Aloe vera* varies with a number of factors, such as annual season, rainfall and temperature, incident solar radiation, harvesting time, climate and land, and cultivation methods [10]. Therefore, it was expected to find differences in the *Aloe vera* composition determined in the present study as compared to other reports. In addition, in this work, the fermentation was carried out with *Aloe vera* extract and not with *Aloe vera* gel, which is another factor that may influence the organic acids detected and the amount obtained.

Although lactic acid has been detected in some samples, lactic acid is not a natural component of *Aloe vera*. Lactic acid determined in *Aloe vera* samples has been assigned as a consequence of microbiological or enzymatic alteration of the product [38]. It has been reported that lactic acid may be produced from *Aloe vera* malic acid by LAB [37]. Accordingly, Zhang et al. [37] did not detect lactic acid in samples of fresh leaves but detected it in commercialized powders. Bozzi et al. [36] determined the concentration of organic acids in *Aloe vera* fresh gel (used as reference material) and in nine commercial *Aloe vera* gel powders. The only organic acid contained in fresh *Aloe vera* gel was malic acid. On the other hand, commercial *Aloe gel* powders contained great concentrations of organic acids other than malic. The most abundant were citric, lactic and succinic acids. However, the presence of lactic and succinic acid was assigned as indicative of bacterial fermentation and enzymatic degradation; while acetic acid present in some samples, even if at low concentrations, was assigned to chemical degradation. To minimize the enzymatic reaction, *Aloe vera* leaves must be processed as quickly as possible after harvesting, or an adequate thermal treatment may be applied [36].

In this work, lactic acid was detected in the *Aloe vera* extracts used for fermentation. Since fresh commercialized non-thermally treated leaves were herein used, its presence is justified. However, it is worth noting that the extract is intended for fermentation with LAB and therefore, this initial lactic acid amount is residual as compared to that obtained at the end of fermentation. Accordingly, during *Aloe vera* fermentation, mainly lactic acid was produced by both *E. faecium* and *L. lactis*, which was expected as they are both LAB (Figure 4). Lactic acid started to be produced in the first 12 h, but it only increased considerably after 24 h fermentation, reaching its maximum production. For *E. faecium*, the maximum amount of lactic acid produced was 11,530 ± 978 mg/L, which represented a 21.6 times increase when compared to its initial concentration (Figure 5a). In the case of *L. lactis*, it produced 6779 ± 998 mg/L of acid lactic (Figure 5b), which was half of the amount produced by *E. faecium* but still represented a 12.7 times increase in its initial content. After 24 h and up to the end of the fermentation (48 h), there was no more production of lactic acid or consumption (*p* < 0.05).

Both bacteria produced also formic acid in the *Aloe vera* juice, although the formic acid started to be consumed after 12 h of fermentation. The maximum amount of formic acid in the juice fermented with *E. faecium* and *L. lactis* was 1812 ± 108 and 1173 ± 105 mg/L, respectively. At the end of fermentation, formic acid was almost totally consumed. There was only 66 ± 7 mg/L of formic acid in the juice fermented by *E. faecium* and 44 ± 4 mg/L by the *L. lactis*. As for lactic acid, *E. faecium* produced more formic acid than *L. lactis*.

Acetic acid did not significantly vary during the fermentation with *E. faecium*, but it slightly decreased during the *L. lactis* fermentation. The acetic acid identified may derive from the degradation of the *Aloe vera* acetylated polysaccharides [37]. The acetic acid produced has been related to *Enterococcus*, *Lactobacillus*, and Bifidobacterium fermentation [39]. Specifically, during *Aloe vera* chemical degradation with LAB, acemannan is deacetylated, resulting in the production of acetic acid [36]. Propionic and iso-butyric acid seem to be also slightly consumed during the fermentation, but their concentration did not vary significantly during fermentation with both bacteria (*p* > 0.05).

The same organic acids were obtained by other authors upon *Aloe vera* fermentation with *Lactobacillus*. Yagi et al. [33] obtained lactic, acetic and propionic acids after 24 h fermentation with *Lactobacillus fermentum* but not butyric acid. Organic acids such as lactic, acetic, propionic and butyric acid were released as secondary products of mahewu non-alcoholic fermented beverage containing *Aloe vera barbadensis* powder [40].

In general, the production of organic acids is related to *Aloe vera* carbohydrates. Carbohydrates are fermented as substrates releasing SCFA as end products, primarily acetate, propionate and butyrate. Acemannan and glucomannan from *Aloe vera* stimulate also an increased production of SCFA by promoting the growth of probiotic colonic bacteria [31,41]. The production of organic acids depends however on factors such as culture medium, growth duration, and the number of available substrates that affect bacterial fermentation [42].

Lactic acid is especially important as it is easily absorbed by the body and hence more often recommended to infants and the elderly [43]. Lactic acid holds antimicrobial and immune-modulating properties. It is probably the main responsible for generating the protective acidic environment that is considered so important for antimicrobial activity [44]. It has been described that in the context of conventional immune cells, L-lactic acid has anti-inflammatory or pro-inflammatory effects depending on the experimental conditions [44,45].

On the other hand, SCFA as acetate promotes a controlled inflammation and avoids pathogen invasion [46]. Propionate, a gluconeogenerator, has been shown to inhibit cholesterol synthesis. Butyrate has been studied for its role in nourishing the colonic mucosa and in the prevention of colon cancer by promoting cell differentiation, cell-cycle arrest, and apoptosis of transformed colonocytes, inhibiting the enzyme histone deacetylase and decreasing the transformation of primary bile acids to secondary bile acids. Overall, SCFA may reduce the risk of developing gastrointestinal disorders, cancer, and cardiovascular disease [47]. Therefore, the produced fermented *Aloe vera* juice holds great potential as a functional drink, with most probably probiotic and prebiotic properties.

### 2.4. Phenolic Compounds and Aloin Quantified during Fermentation

**Figure 6:**
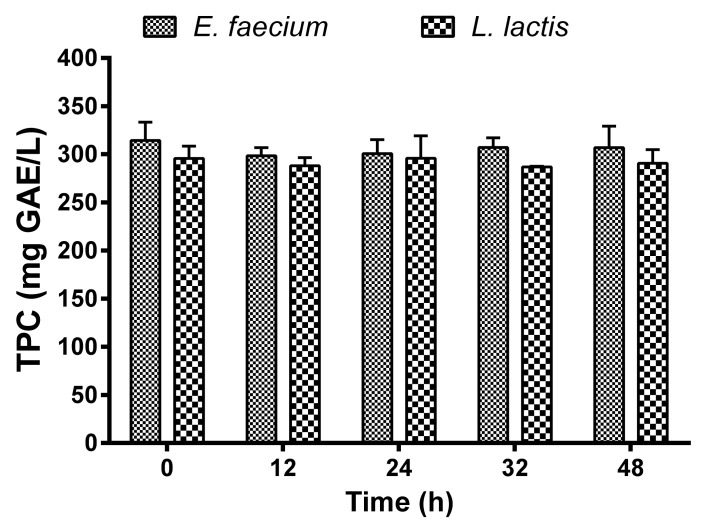
Total phenolic content (TPC) during the fermentation of *Aloe vera* extract with *Enterococcus faecium* and *Lactococcus lactis*.

Figure 6 shows the TPC obtained in the *Aloe vera* juice during the 48 h fermentation with *E. faecium* and *L. lactis.* The initial concentration of TPC in the fermented extract with *E. faecium* and *L. lactis* was 295.72 ± 13.2 and 314.30 ± 26.2 mg GAE/L, respectively. Our results showed no significant differences in the TPC content along the fermentation using both probiotic bacteria (*p* > 0.05). In fact, an important matter is that *E. faecium* and *L. lactis* did not change the amount of TPC from the *Aloe vera*. No significant differences were found in the final TPC obtained in fermentations inoculated either with *E. faecium* or *L. lactis*.

In general, the microbial metabolism of phenolic compounds is influenced by the composition of the raw material (plant-based foods) and by the fermentation conditions (microorganisms, time, temperature, among others). Therefore, synergistic effects that may occur from interactions with other compounds present in the raw material will depend on the phenolic compound source. In the present study, no significant changes occurred in the TPC during fermentation, similarly to what was obtained by Liu et al. [48] in a tomato juice fermentation by *Lactobacillus casei* and *Lactobacillus* *plantarum*. However, some studies have demonstrated that LAB strains can degrade certain phenolic compounds into other metabolites, which may even exert higher biological activities than their precursors [49,50,51].

The profile of the individual biocompounds was tentatively determined during fermentations. The initial chemical profile of the juice (T0) was similar for the two fermentations (see Table 1). Fourteen compounds were identified in the *Aloe vera* extract, mostly flavonoids (naringin, hesperidin, kaempferol, epicatechin, quercetin and taxifolin), some hydroxycinnamic acids (ferulic, chlorogenic and p-coumaric acids), hydroxybenzoic acids (vanillic, 3,4 dihydroxybenzoic and ellagic acids), stilbene (resveratrol), and anthraquinone (aloin) (Table 1).

Epicatechin was the compound detected at the highest quantity in the initial *Aloe vera* juice (≈48 mg/L), followed by aloin (≈27 mg/L), hesperidin (≈16 mg/L) and ellagic acid (≈15 mg/L). The other phenolic compounds were quantified in concentrations lower than 10 mg/L.

Most of these compounds have already been previously identified by other authors in *Aloe vera* [52,53,54,55]. Quispe et al. [54] identified aloesin, chlorogenic acid, caffeic acid, *Aloe* emodin-diglucoside, isoquercitrin, and kaempferol, by UHPLC and Orbitrap high-resolution mass spectrometry. Sumi et al. [55] determined good amounts of phenolic antioxidants, such as procyanidins (epicatechin and catechin), quercetin and phenolic acids (caffeic, ferulic, ellagic and vanillic) in *Aloe vera* hydroalcoholic extracts.

*Aloe vera* can be an important dietary source of bioactive compounds that contribute to health-span improving life quality, demonstrating their potential as nutraceutical, functional foods and/or components for therapeutic purposes. The vast pharmacological properties of *Aloe vera* arise from its various chemical constituents including polysaccharides, anthraquinones, and phenolics. For example, epicatechin, an epimer of catechin, has shown multiple therapeutic benefits, including heart disease prevention, as well as anticancer and antibacterial effects, and it is an excellent antioxidant [56,57]. Epicatechin acts as an antioxidant both directly, as a scavenger of free radicals, and indirectly, as a modulator of superoxide dismutase and glutathione peroxidase [58]. Kaempferol, another natural flavonoid present in *Aloe vera*, has been related to antimicrobial, antiviral, anti-inflammatory, antioxidant, antitumor, cardioprotective, neuroprotective and antidiabetic activities [59,60], as well as provides beneficial effects on the tight junction barrier integrity of intestinal Caco-2 cell monolayers [61]. Aloin is an important anthraquinone compound for the pharmaceutical industry, since it is used in the preparation of diacerein, which is a drug used for treating osteoarthritis [62]. Aloin has been reported in *Aloe barbadensis* Miller, *Aloe arborescens* and *Aloe grandidentata* [63]. Aloin has antioxidant properties, even at low concentration [64]. Therefore, it has been used to lower oxidative stress indices in diseases such as cancer, diabetes, and other cardio-metabolic diseases [10,55,65,66,67].

During *Aloe vera* fermentation with either *E. faecium* or *L. lactis*, the individual profile of the phenolic compounds and anthraquinone slightly changed (see Table 1). During fermentation with *E. faecium*, the initial aloin content decreased by about 12.4% and naringin increased by approximately 18.5%. Some compounds that decreased their content during fermentation with *L. lactis* were naringin, hesperidin, quercetin and taxifolin; kaempferol increased by about 32.9%.

The content of epicatechin, resveratrol, quercetin and chlorogenic, 3,4-dihydroxybenzoic, and ferulic acids have not changed during the fermentation of *Aloe vera* with *E. faecium*. Meanwhile, for *L. lactis* fermentation, vanillic, chlorogenic, coumaric, ellagic, 3,4-dihydroxybenzoic and ferulic acid, including resveratrol, were not altered.

Overall, *Aloe vera* fermented with *E. faecium* maintains its content in total individual phenolic compounds analyzed (146 ± 11 and 137 ± 15 mg/L, at 0 and 48 h, respectively). However, when the *Aloe vera* is fermented with *L. lactis*, a slight decrease is observed (150.61 ± 3.42 to 118.30 ± 2.86 mg/L), as shown in Table 1. Possibly, these compounds were metabolized by bacteria.

Hydroxybenzoic and hydroxycinnamic acids can be decarboxylated by LAB to the corresponding phenolic or vinyl derivatives or hydrogenated by phenolic acid reductases. *Lactobacillus rossiae*, *Lactobacillus Brevis* and *Lactobacillus curvatush* strains have followed one of the two pathways (decarboxylation or reduction) and can metabolize phenolic compounds (coumaric and ferulic acids) into their vinyl derivatives. In contrast, *Leuconostoc mesenteroides* and *Lactobacillus fermentum* strains were not able to metabolize hydroxycinnamic acids because of the enzymes involved in metabolism [68]. This may justify why both bacteria did not metabolize ferulic acid during fermentation. An increase in ferulic acid has been reported for bread fermentation with baker’s yeast [69].

One of the factors influencing the reduction of phenolic compounds is microbial fermentation. The microorganisms have specific metabolic activities depending on the strain or species of microorganism and the enzyme portfolio. On the other hand, phenolic compounds will also influence the growth of these bacteria, and some of them will be degraded by LAB as a consequence of their growth [68]. However, limited studies have been performed on the influence of phenolic compounds on the growth and viability of LAB species. In one study, the effect of hydroxycinnamic acids, their quinic esters and quinic acid (a non-phenolic acid) on the growth of *L. plantarum* was evaluated [70]. It was shown that bacterial growth was only affected by hydroxycinnamic acid at concentrations up to 3 mM.

Filannino et al. [49] reported no changes in the concentration of rutin, quercetin, chlorogenic acid, neochlorogenic acid and *p*-coumaroylquinic acid during the lactic fermentation process of a cherry juice. However, most tested LAB strains changed the concentration of phenolic acids. *L. plantarum* decreased the concentration of protocatechuic acid by about 70% and consumed *p*-coumaric acid.

Other authors have reported that the growth of LAB, specifically *Oenococcus oeni*, was affected by phenolic compounds in different ways, depending on their type and concentration. Overall, there was no effect at low concentrations, but hydroxycinnamic acids were inhibitory at high concentrations [51].

### 2.5. Antioxidant Activity during Fermentation Process

**Figure 7:**
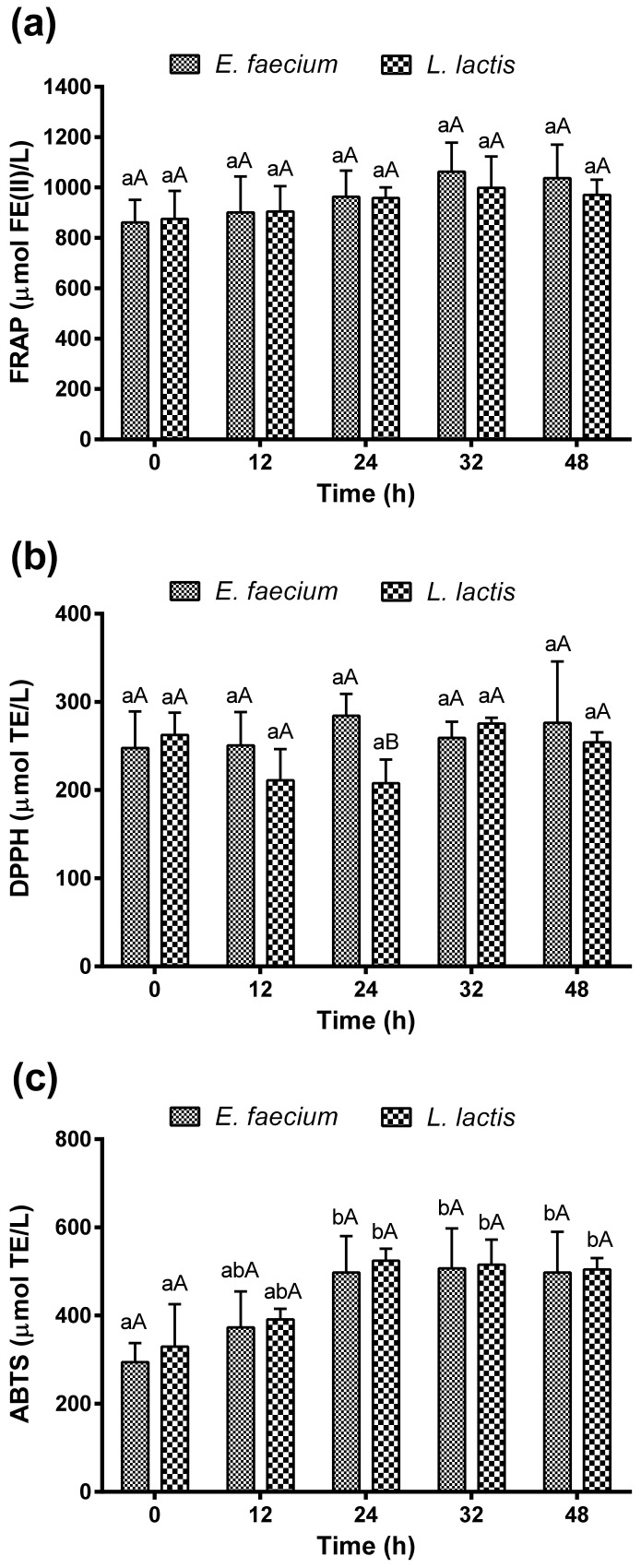
Antioxidant activity during fermentation with *Enterococcus faecium* and *Lactococcus lactis* by FRAP (**a**), DPPH (**b**), and ABTS (**c**). Different lowercase letters a, b show significant differences (*p* < 0.05) between different fermentation times for the same bacteria. Different uppercase letters A, B show significant differences (*p* < 0.05) between bacteria for the same fermentation time.

The antioxidant activity determined during fermentation with *E. faecium* and *L. lactis* is shown in Figure 7. Three antioxidant assays were used to evaluate the antioxidant capacity of the fermented *Aloe vera* juice—one method based on metals reduction (FRAP) and two methods based on chemical aspects, measuring the radical scavenging activity (DPPH^•^ and ABTS^•+^).

For the FRAP assay, the antioxidant capacity slightly increased from 861 ± 68 to 1038 ± 116 µmol Fe (II)/L for *E. faecium* (36 h) and 875 ± 78 to 970 ± 48 µmol Fe (II)/L (48 h) for *L. lactis*. However, no statistically significant differences were found during the *Aloe vera* fermentation, even between the fermentations run with the different bacteria (*p* > 0.05) (Figure 7a).

For the DPPH assay, the initial free radical scavenging activity of the *Aloe vera* fermented by *E. faecium* and *L. lactis* was determined as 247 ± 34 µmol TE/L and 262 ± 19 µmol TE/L, respectively. The antioxidant activity was constant during fermentation with *E. faecium*. For *L. lactis*, it slightly decreased in the first 24 h fermentation (207 ± 27 µmol TE/L), but it further increased at 36 h (275 ± 8 µmol TE/L) without significant variation throughout the fermentation (Figure 7b).

The ABTS method showed an increase in the antioxidant activity of *Aloe vera* throughout the fermentation with both LAB. For *E. faecium*, there was an observed increase from 324 ± 60 to 497 ± 63 µmol TE/L (24 h, *p* < 0.05) and for *L. lactis* from 329 ± 69 to 524 ± 19 µmol TE/L (24 h, *p* < 0.05). There were no significant differences in antioxidant activity between fermentation with *E. faecium* and *L. lactis*, as both bacteria follow the same trend during fermentation (Figure 7c).

The concentrations of different antioxidant polyphenols are not the only factor influencing antioxidant capacity of the *Aloe vera* juice, as the individual antioxidant activity of phenolic compounds may differ when acting in synergy with each other, having different mechanisms of action. The structural arrangements of these compounds (number and position of hydroxyl groups, double bonds and aromatic rings) play also an important role [22]. Additionally, their contributions to FRAP, DPPH and ABTS may differ due to the sensibility of each method.

Differences between the antioxidant activities reported for *Aloe vera* may be related to factors that influence its chemical composition, such as different existing varieties of *Aloe vera*, annual season rainfall and temperature, incident solar radiation, harvesting time, climate and cultivation methods [10] as well as, by the methods used for the extraction of the bioactive compounds, in this case, water plus cellulase.

The content on phenolic compounds in *Aloe vera*, and in other raw materials, is closely correlated with its antioxidant activity [24]. The antioxidant activity of an *Aloe vera* gel in fresh weight was determined as 0.34 ± 0.01 mM TE/g for DPPH assay, 2.06 ± 0.06 mM ET/g by ABTS, and 0.38 ± 0.01 ET/g FM by FRAP, sustaining the high antioxidant potential of *Aloe vera* [54]. Accordingly, similar antioxidant activity was determined in the present work, independently of the LAB applied, which is maintained or slightly increased during fermentations.

The increase in antioxidant activity of *Aloe vera* juice can be influenced by the metabolism of phenolic compounds, some organic acids and sugars, which have antioxidant capacity. This means that the increase in the antioxidant activity observed is probably associated to the organic acids produced during fermentation, or it is a result of the synergistic effect between the bioactive compounds of the *Aloe vera*, namely, the phenolic compounds, SCFA and sugars. The same was suggested by Mazzulla et al. [71] in a work conducted with an aqueous extract of *Aloe vera* leaves.

To the best of our knowledge, the evaluation of the antioxidant activity of *Aloe vera* fermented with *E. faecium* and *L. lactis* has not been studied so far. A similar trend in the antioxidant activity was found for tomato fermented with *L. plantarum* and *L. casei* [48]. An increase in the antioxidant activity was determined by the three methods evaluated (FRAP, DPPH and ABTS) as the fermentation progressed. In another work, the fermentation of goji berry juice by different mixtures of multiple bacterial strains, including LAB, has improved significantly the antioxidant capacity of the juice, which was strongly correlated with the content of the free forms of phenolics [72]. However, no obvious difference was found in the DPPH free radical scavenging rates among the fermentations run with the different bacterial mixtures. Our results agreed in that there were no significant differences between fermentations with the different LAB (*E. faecium* and *L. lactis*).

*Aloe vera* antioxidant activity is mainly related with compounds such as aloin, coumarin, flavonoids and other kind of phenolic compounds. Several authors have associated *Aloe vera* antioxidant activity to isoaloeresin D, 8-C-β-d-[2-O-(E)-coumaroyl], glucopyranosyl-2-[2-hydroxy]propyl-7-methoxy-5-methylchromone and aloe dihydroisocoumarin [73,74,75]. However, there are other compounds with antioxidant activity, such as indoles and alkaloids, which were not identified in this study and could interfere with the quantified antioxidant activity.

Some *Aloe vera* polysaccharides, rhamnose and arabinose, and ascorbic acid have also been assigned as compounds with antioxidant potential [10,76,77,78]. In this work, *Aloe vera* extracts used for fermentation contained cellulase (used in the extraction), which enzymatically hydrolyzed the sugars. This might have improved the availability and stability of other antioxidant compounds such as phenolics and proteins and contributed to the improved antioxidant activity of the final juice [10].

Phenolic compounds in foods originate from one of the main classes of secondary metabolites in plants [79]. At a low concentration, phenolics act as an antioxidant and protect food from oxidative rancidity [80]. Phenolic antioxidants interfere with the oxidation process as free radical terminators and sometimes also as metal chelators. Phenols have been widely studied and confirmed to possess diverse bioactivities, which could be beneficial to human health [24]. In this sense, their consumption and presence in the organism are important since they are not produced by humans. Fermented *Aloe vera* juice can be an interesting source of bioactive compounds (mainly antioxidants, prebiotics, and probiotics), promoting consumers’ health.

## 3. Materials and Methods

### 3.1. Chemicals

Cellulase from thrichoderma, Folin–Ciocalteu phenol reagent, 2,2-diphenyl-1-picrylhydrazyl (DPPH), 2,2′–azinobis-(3-ethylbenzothiazoline-6-sulfonate) (ABTS), 2,4,6-tris(2-pyridyl)-S-triazine (TPTZ), gallic acid, glucose, 6-hydroxy-2,5,7,8-tetramethylchroman-2-carboxylic acid (Trolox), vanillic acid, chlorogenic acid, catechin, epicatechin, *p*-coumaric acid, ellagic acid, naringin, hesperidin, resveratrol, ferulic acid, quercetin, 3,4-dihydroxybenzoic, taxifolin, aloin, kaempferol, lactic, acetic, formic, propionic, iso-butyric, n-butyric and valeric acid, glucose, fructose, arabinose, mannose and galactose, ferric chloride hexahydrate, sodium acetate trihydrate, glacial acetic acid, hydrochloric acid and sodium acetate trihydrate were purchased from Sigma-Aldrich Ltd. (St. Louis, MO, USA). All other chemicals used were of analytical grade, and water was ultra-pure.

### 3.2. Aloe vera Gel Preparation

Four-year-old fresh whole leaves of *Aloe barbadensis* Miller (*Aloe vera*), harvested from Ibiza plantations and commercialized by Ulíavera (Córdoba, Spain), were washed with water, immersed in a 2.0% sodium hypochlorite solution, and rinsed with distilled water. For each leaf, the spikes, inferior, and superior parts were removed before longitudinally slicing to separate the epidermis from the parenchyma (gel). Then, the gel was pressed by means of a laboratory manual roll processor, and next, it was lyophilized and stored at −20 °C until further analyses were performed.

### 3.3. Aloe vera Extract Preparation

The powdered gel of *Aloe vera* (1 g) underwent four different solid–liquid extractions to recover its bioactive compounds. The following solvents were tested: (1) water; (2) water plus enzyme (Cellulase 45 mg); (3) ethanol: water (80:20 (*v*/*v*)); (4) ethanol: water (80:20 (*v*/*v*)) plus enzyme (Cellulase 45 mg). The volume of extraction applied in all experiments was 30 mL. Experiments were carried out for 1 h at 45 °C using 100 mL cylindrical reactors duly protected from light in a thermostatic water bath with shaking (150 rpm). The supernatants were then filtered through Whatman No. 4 filter paper, and the insoluble fibers were removed.

The solvent efficiency in extracting target compounds from a dry *Aloe vera* gel can be measured using the extraction yield. Yield (presented in percentage) was calculated using Equation (1), considering the cumulative mass of extract:

(1) $$\mathrm{Yield}=\frac{\mathrm{extracted}\;\mathrm{solids}\;\left(g\right)}{\mathrm{initial}\;\mathrm{dry}\;\mathrm{material}\;\left(g\right)}\times 100$$

Finally, the *Aloe vera* extracts were sterilized by filtration with an acetate cellulose 0.22 µm membrane. The phytochemical profile of the extracts was determined in terms of carbohydrates and phenolic compounds. The extract with the best profile was selected for further fermentation with the probiotic bacteria (Section 3.6).

### 3.4. Screening of Bioactive Compounds in the Aloe vera Juice

#### 3.4.1. Carbohydrates Analysis

Monosaccharides from the *Aloe vera* gel, namely, glucose, fructose, mannose, galactose and arabinose, were determined by high-performance liquid chromatography (HPLC) using a Varian HPLC system (Agilent Technologies 1260 Infinity 2) equipped with a Metacarb 87H column (0.78 cm × 30 cm). Samples were eluted at a flow rate of 0.7 mL/min and 60 °C with a mobile phase of H_2_SO_4_ (0.005 M). Quantification was carried out using calibration curves for each compound and analyzed at concentrations between 500 and 100 mg/L. In all cases, the coefficient of linear correlation was R^2^ > 0.99. The results were expressed in milligram per liter of *Aloe vera* juice (mg/L).

#### 3.4.2. Total Phenolic Content Analysis

The total phenolic content (TPC) was determined using a 96-well microplate colorimetric assay by the Folin–Ciocalteu method adapted from Ferreira-Santos et al. [81]. Briefly, 10 µL of extraction samples were mixed with 60 µL of Na_2_CO_3_ (75 g/L), 15 µL of Folin–Ciocalteu reagent and 200 µL of ultra-pure water. The mixture was incubated at 60 °C for 5 min under agitation (150 rpm). The absorbance was measured using a spectrophotometer (Synergy HT, Biotek Instruments, Inc., Winooski, VT, USA) at λ = 700 nm. A calibration curve was prepared using a standard solution of gallic acid (1500–50 mg/L, R^2^ = 0.99), and the results were expressed in mg of gallic acid equivalents (GAE) per liter of *Aloe vera* juice (mg/L).

### 3.5. Inoculum Preparation

Two probiotic bacteria, namely an *Enterococcus* and a *Lactococcus*, were used in this work. In a previous work of the research group, bacteria were isolated from human breast milk and further identified as *Enterococcus faecium*. *Lactococcus lactis* BS-10 by Chr Hansen was used as a control, since it is a commercialized probiotic bacterium. Strains were reactivated in Man Rogosa Sharpe (MRS) broth and incubated at 37 °C for 48 h. The inoculum was prepared by means of the MC Farland 0.5 scale, which was adjusted to 1 × 10^6^ CFU/mL.

To guarantee the efficient growth of *E. faecium* and *L. lactis* in a culture medium based on *Aloe vera*, a preliminary assay was carried out to assess possible growth inhibition by the *Aloe vera* juice. An inoculum of 1 × 10^6^ CFU/mL of *E. faecium* and *L. lactis* was prepared and seeded on MRS agar plates, separately. A volume of 50 μL of the juice were placed in wells made in the MRS agar plates. Ampicillin (50 mg/mL) was used as a positive control, and water was used as a negative control. A bacterial strain was defined as susceptible or resistant when its growth was inhibited or not, respectively. After 24 h, at 37 °C, the agar plates were observed and images were captured using a ChemiDoc Imaging System (Bio-Rad Laboratories, Inc., Hercules, CA, USA).

### 3.6. Fermentation Kinetics of the Aloe vera Juice with the Probiotics

Fermentation was run with the extract that obtained better chemical composition (Section 3.2).

For the fermentation, 100 mL of the previously selected *Aloe vera* extract (Section 3.2) was inoculated for 48 h at 37 °C with *E. faecium* and *L. lactis* (1400 µL at 1 × 10^6^ CFU/mL), respectively. During fermentation, 5 mL of samples were collected every 12 h for evaluation of the pH (measurements at 25 °C using a digital pH meter (HANNA)), the optical density (by spectrophotometry, at λ = 540 nm (Synergy HT, Biotek Instruments, Inc.)) and the bioactive compounds, namely, carbohydrates (see Section 3.4.1), organic acids (see Section 3.6.1), phenolic compounds (TPC, see Section 3.4.2 and individual content, see Section 3.6.2) and its antioxidant activity (see Section 3.6.3).

#### 3.6.1. Organic Acids Analysis

Organic acids were analyzed by HPLC using a Varian HPLC system (Agilent Technologies 1260 Infinity 2) equipped with a Rezex ROA–Organic Acid H^+^ column (300 mm × 7.8 mm) working at 60 °C. A mobile phase of H_2_SO_4_ (2.5 mM) was used to elute the samples at a flow rate of 0.6 mL/min. Calibration curves of ten volatile fatty acids (VFA) were designed (lactic, formic, acetic, propionic, iso-butyric, n-butyric and valeric acid) at concentrations ranging between 30 and 2500 mg/L (R^2^ = 0.99). Results were expressed in milligrams per liter (mg/L).

#### 3.6.2. Phenolic Compounds Analysis

Individual compounds were identified and quantified by Ultra-Performance Liquid Chromatography (UPLC) as described and validated in [81,82]. A Shimadzu Nexpera X2 UPLC chromatograph equipped with a Diode Array Detector (DAD) (Shimadzu, SPD-M20A, Columbia, MA, USA) was used. Separation was performed at 40 °C on a reversed-phase Aquity UPLC BEH C18 column (2.1 mm × 100 mm, 1.7 µm particle size; from Waters, Milford, MA, USA) equipped with a pre-column of the same material. Samples were eluted with two HPLC grade solvents, water/formic acid (0.1%) and 100% acetonitrile, at a flow rate of 0.4 mL/min. Biocompounds were identified by comparing their UV spectra and retention times with those of the corresponding standards. Calibration curves were drawn for a range of concentrations between 250 and 2.5 mg/mL per compound analyzed (vanillic acid, chlorogenic acid, catechin, epicatechin, *p*-coumaric acid, ellagic acid, naringin, hesperidin, resveratrol, ferulic acid, quercetin, 3,4-dihydroxybenzoic, taxifolin, aloin and kaempferol (R^2^ > 0.99)). Compounds were quantified and identified at different wavelengths (209–370 nm). Results were expressed in milligrams per liter (mg/L).

#### 3.6.3. Antioxidant Activity Analysis

The antioxidant activity was determined using the following methods: ferric reducing antioxidant power (FRAP assay), 2,2-difenil-1-picrylhydrazyl (DPPH assay) and 2,2′-azino-bis-(3-ethylbenzothiazoline-6-sulfonic acid) (ABTS assay) by scavenging activity mechanism.

##### Ferric Reducing Antioxidant Power (FRAP)

FRAP assay was performed according to the method described by [83], with few modifications. A 10 µL sample (properly diluted and filtered) was mixed with 290 µL of FRAP reagent in a 96-well microplate. The resultant reaction mixture was incubated at 37 °C for 15 min. Next, absorbance was determined at λ = 593 nm in a spectrophotometric microplate reader (Synergy HT, Biotek Instruments, Inc.) against a blank prepared with water. A calibration curve was prepared, using an aqueous solution of FeSO_4_.7H_2_O (200, 400, 600, 800 and 1000 µM). FRAP values were expressed as micromoles of ferrous equivalent per liter of *Aloe vera* juice (µmol Fe (II)/L).

##### DPPH Assay

The DPPH radical scavenging activity was determined using the method described by Ballesteros et al. [84]. The reaction was carried out in a 96-well microplate containing 25 µL of the sample (fermented or not) and 200 µL of 150 µM 2,2-diphenyl-1-picrylhydrazyl (DPPH) solution (dissolved in 80% methanol to an absorbance of 0.70 ± 0.01 at λ = 515 nm). Solutions were vortexed and allowed to stand for 1 h in the dark, until complete reaction, at room temperature. Furthermore, the absorbance was measured at λ = 515 nm in a spectrophotometric microplate reader (Synergy HT, Biotek Instruments, Inc.), using water as blank. A calibration curve was prepared with a standard solution of Trolox (6-hydroxy-2,5,7,8-tetramethylchroman-2-carboxylic acid) diluted in methanol (40, 80, 100, 300, and 400 µM). DDPH percent inhibition data were plotted as a function of antioxidant concentration to obtain DPPH inhibition concentration at 50% (IC_50_). The IC_50_ values were expressed as micromoles of Trolox equivalent (TE) per liter of *Aloe vera* juice (µmol TE/L).

##### ABTS Assay

The radical cation decolorization (ABTS) assay was performed according to the method described by Ballesteros et al. [84] with few modifications. ABTS solution (7 mM) and potassium persulfate (2.45 mM) were mixed in a 1:1 ratio. The reaction occurred in the dark for 12–16 h to produce the ABTS^•+^ cation radical solution. A stock solution was then diluted with 80% methanol solution, up to an absorbance of 0.700 ± 0.020, determined at λ = 734 nm. Briefly, 10 μL of samples were mixed with 200 μL of ABTS^+^ working solution in a 96-well microplate. The plate was then incubated at 37 °C for 15 min in a microplate reader (Synergy HT, Biotek Instruments, Inc.). The absorbance was then read at λ = 734 nm. A calibration curve was drawn using a standard solution of Trolox diluted in ethanol (50, 100, 200, 250, 300, 400 and 500 µM). ABTS percent inhibition data were plotted against antioxidant concentration to obtain the ABTS inhibition concentration at 50% (IC_50_). The IC_50_ values were expressed as micromoles of Trolox equivalent (TE) per liter of *Aloe vera* juice (µmol TE/L).

### 3.7. Statistical Analysis

Analyses were carried out on three independent replicates. Each replicate was analyzed twice. Data were subjected to one-way ANOVA; pair-comparison of treatment means was obtained by Tukey’s procedure at *p* < 0.05, using the Statistical software GraphPad Prism^®^ (version 5.0; San Diego, CA, USA).

## 4. Conclusions

The biotransformation of bioactive compounds from the *Aloe vera* juice was assessed during fermentation with *E. faecium* and *L. lactis*. *Aloe vera* demonstrated to be a feasible medium for the growth of *E. faecium* and *L. lactis*. Both LAB strains used the *Aloe vera* sugars as a carbon source and produced mainly lactic acid. The content of total phenolics and SCFA of the *Aloe vera* juice was not affected during fermentation, and the antioxidant activity tended to increase. This synergy between *Aloe vera*—as a prebiotic, antioxidants and source of other bioactive compounds, and LAB—as probiotics, may be an excellent basis for the development of a functional commercial *Aloe vera* beverage with great benefits for human health.

## Figures and Tables

**Table 1:** Individual phenolic compounds and aloin determined in the *Aloe vera* juice during fermentation by *Enterococcus faecium* and *Lactococcus lactis*.

Compounds (mg/L)	0 h		12 h		24 h		36 h		48 h	
	*E. faecium*	*L. lactis*	*E. faecium*	*L. lactis*	*E. faecium*	*L. lactis*	*E. faecium*	*L. lactis*	*E. faecium*	*L. lactis*
Vanillic acid	2.05 ± 0.13	2.21 ± 0.18	1.56 ± 0.41	2.34 ± 0.12	1.87 ± 0.10	2.32 ± 0.03	n.d.	1.48 ± 0.06	n.d.	1.24 ± 0.07
Chlorogenic acid	0.73 ± 0.02	0.64 ± 0.04	0.48 ± 0.02	0.59 ± 0.13	0.85 ± 0.00	0.75 ± 0.01	0.72 ± 0.01	0.91 ± 0.14	0.74 ± 0.03	0.64 ± 0.03
Epicatechin	45.25 ± 5.63	51.15 ± 0.68	40.05 ± 2.91	54.82 ± 5.98	43.41 ± 2.07	45.90 ± 5.67	43.49 ± 0.22	38.08 ± 1.71	44.62 ± 4.88	39.28 ± 0.49
*p*-Coumaric acid	2.32 ± 0.41	1.93 ± 0.08	1.83 ± 0.33	2.25 ± 0.28	1.58 ± 0.03	1.84 ± 0.13	0.83 ± 0.36	1.48 ± 0.16	1.74 ± 0.30	1.43 ± 0.05
Ellagic acid	15.98 ± 1.47	14.79 ± 0.12	17.07 ± 0.63	15.68 ± 0.12	15.78 ± 1.13	13. 80 ± 1.36	12.44 ± 1.88	15.73 ± 1.92	14.06 ± 0.17	14.26 ± 0.08
Naringin	7.95 ± 0.28	7.86 ± 0.36	8.64 ± 1.15	8.47 ± 0.46	9.67 ± 3.23	7.10 ± 0.05	9.14 ± 0.00	5.15 ± 0.01	9.42 ± 2.30	6.73 ± 0.18
Hesperidin	16.80 ± 1.83	15.02 ± 0.35	14.58 ± 1.47	16.46 ± 1.24	13.32 ± 0.00	14.62 ± 0.58	10.12 ± 1.60	13.01 ± 0.72	18.70 ± 5.84	12.79 ± 0.20
Resveratrol	4.67 ± 0.10	4.77 ± 0.14	4.50 ± 0.04	4.83 ± 0.15	4.43 ± 0.11	4.72 ± 0.05	4.34 ± 0.14	4.29 ± 0.27	4.14 ± 0.03	4.18 ± 0.03
Ferulic acid	3.29 ± 0.17	3.49 ± 0.06	5.53 ± 1.79	3.80 ± 0.07	3.46 ± 0.01	3.08 ± 0.78	3.76 ± 0.38	5.02 ± 1.00	2.91 ± 0.05	3.18 ± 0.24
Quercetin	7.60 ± 0.29	9.10 ± 0.57	1.51 ± 0.48	1.23 ± 0.21	7.57 ± 0.53	1.82 ± 0.79	6.68 ± 0.02	1.79 ± 0.79	6.37 ± 0.35	1.04 ± 0.01
3,4-Dihydroxybenzoic acid	0.31 ± 0.01	0.31 ± 0.04	0.41 ± 0.01	0.38 ± 0.06	0.62 ± 0.08	0.20 ± 0.08	0.35 ± 0.02	0.18 ± 0.01	0.31 ± 0.09	0.22 ± 0.03
Taxifolin	9.47 ± 0.22	9.49 ± 0.25	8.83 ± 0.25	10.12 ± 0.47	8.51 ± 0.29	8.07 ± 0.89	7.33 ± 0.24	7.67 ± 0.00	7.62 ± 0.39	6.92 ± 0.26
Kaempferol	2.50 ± 0.08	2.43 ± 0.05	2.43 ± 0.08	2.58 ± 0.07	2.35 ± 0.19	2.40 ± 0.03	1.92 ± 0.22	2.58 ± 0.42	2.02 ± 1.03	3.32 ± 1.18
Aloin	27.26 ± 0.50	27.42 ± 0.50	25.99 ± 0.71	29.04 ± 0.98	26.34 ± 1.64	27.07 ± 0.11	22.13 ± 1.69	25.24 ± 4.01	24.03 ± 1.14	23.87 ± 0.97
Total	146 ± 11	150 ± 3	133 ± 10	152 ± 10	140 ± 9	134 ± 11	137 ± 15	123 ± 11	137 ± 15	118 ± 3

Values of phenolic compounds are expressed as concentration mean ± SD (mg/L) of three experiments. n.d.: not detected.

## Data Availability

Not applicable.

## Sample Availability

Samples of the compounds are available from the authors.
